# [3-Meth­oxy-5-(meth­oxy­carbon­yl)isoxazol-4-yl](4-meth­oxy­phen­yl)iodo­nium 2,2,2-tri­fluoro­acetate

**DOI:** 10.1107/S2414314623003000

**Published:** 2023-04-06

**Authors:** Mohd Abdul Fatah Abdul Manan, David B. Cordes, Alexandra M. Z. Slawin, David O’Hagan

**Affiliations:** a Faculty of Applied Sciences, Universiti Teknologi MARA, 40450 Shah Alam, Selangor, Malaysia; b EaStCHEM School of Chemistry, University of St Andrews, North Haugh, St Andrews, KY16 9ST, United Kingdom; Sunway University, Malaysia

**Keywords:** crystal structure, isoxazole, 4-meth­oxy­phen­yl, iodo­nium, dimer

## Abstract

A new isoxazole-based iodo­noium salt, C_13_H_13_INO_5_^+^·C_2_F_3_O_2_^−^, has been synthesized and structurally characterized. In the crystal, ions are linked by short I⋯O contacts to form a neutral tetra-ion aggregate. These combine with C—H⋯F and C—H⋯O inter­actions to form double-layered two-dimensional sheets in the (001) plane.

## Abstract 2

The crystal structure of an unsymmetrical diaryl iodo­nium salt obtained from the nucleophilic coupling of tribut­yl(4-meth­oxy­phen­yl)stannane with a di­acet­oxy­iodo precursor bearing an isoxazole moiety is described.

## Graphical abstract

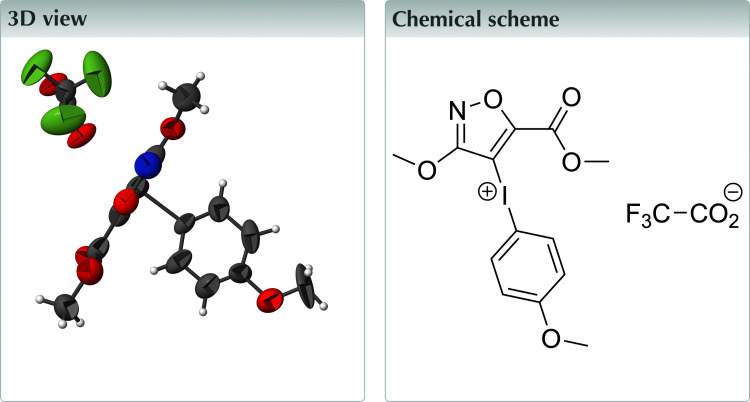

## Structure description

Hypervalent iodine compounds exhibit attractive features of low cost, mild and selective reagents in organic synthesis (Wirth, 2005 ▸; Richardson & Wirth, 2006 ▸). These reagents serve as environmentally benign alternatives to toxic heavy-metal-based oxidants and expensive organometallic catalysts (Satam *et al.*, 2010 ▸; Wirth, 2001 ▸). The application of iodo­nium reagents in organic transformation encompasses areas such as C—C, C–heteroatom and heteroatom–heteroatom bond formation, oxidations, rearrangements and radical reactions (Frigerio & Santagostino, 1994 ▸; Zhdankin & Stang, 2008 ▸; Zhdankin, 2009 ▸, 2011 ▸).

A particularly important application is the reaction of di­aryl­iodo­nium salts with fluorine anions, allowing the introduction of fluorine into chemical compounds of inter­est (Tredwell & Gouverneur 2012 ▸; Tredwell *et al.*, 2008 ▸). Furthermore, by using di­aryl­iodo­nium salts, both electron-deficient and electron-rich rings can be fluorinated, allowing access to all regioisomers of a particular arene over standard S_N_Ar chemistry (Shah *et al.*, 1998 ▸). Moreover, these types of reaction typically require milder conditions than standard S_N_Ar reactions, and they can even take place in wet solvents (Chun *et al.*, 2013 ▸). Features which are privileged for the incorporation of radioactive [^18^F]-fluoride into radiotracer mol­ecules established for Positron Emission Tomography.

The versatility of isoxazoles core components in biologically active compounds, natural products and functional materials (Abdul Manan *et al.*, 2017 ▸; Frolund *et al.*, 2002 ▸; Lee *et al.*, 2009 ▸) led us to examine the synthesis of iodo­noum salts bearing an isoxazole motif possessing novel structural features with the possibly of some inter­est as a precursor to fluoro­isoxazole.

**Figure 1:**
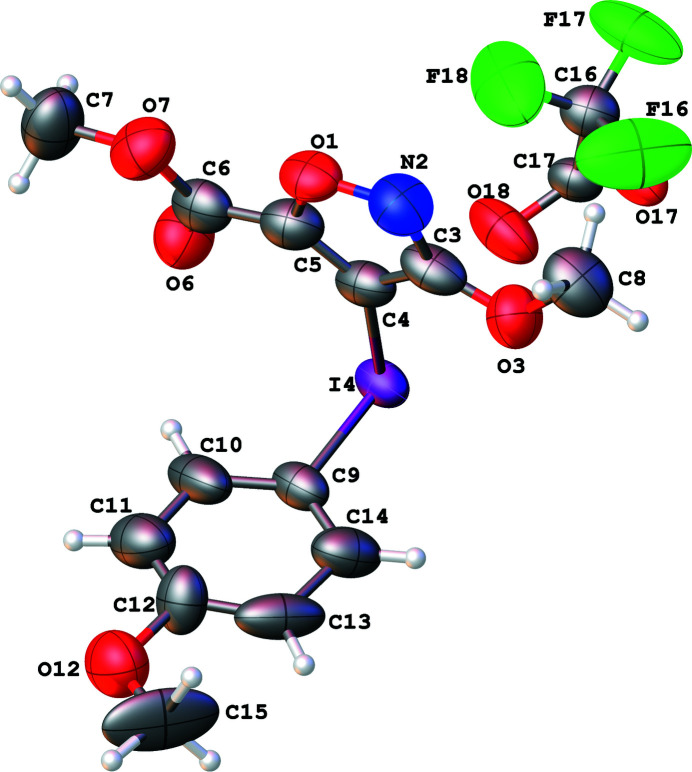
Mol­ecular structures of the constituents of the asymmetric unit of the title compound, showing the atom-labelling scheme and displacement ellipsoids drawn at the 50% probability level.

**Figure 2:**
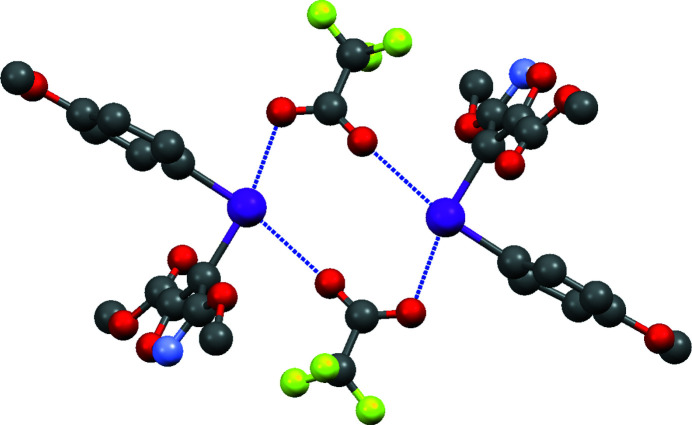
View down the [110] axis of the neutral, tetra-ion aggregate formed by I⋯O inter­actions, shown as dashed lines. Hydrogen atoms are omitted for clarity.

**Figure 3:**
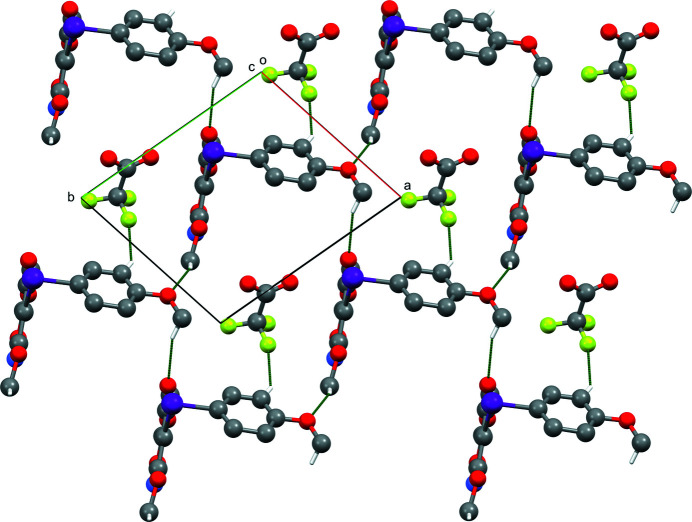
View down the [001] axis of the two-dimensional sheet formed by weak hydrogen-bonding inter­actions, shown as dashed lines. Hydrogen atoms not involved in hydrogen bonding are omitted.

The title salt, C_13_H_13_INO_5_^+^·C_2_F_3_O_2_^−^, crystallizes in the space group *P* 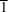 with one ion pair in the asymmetric-unit (Fig. 1 ▸). In the crystal, the ring of the isoxazole group is inclined to the meth­oxy­phenyl ring at an angle of 84.4 (3)° and the C—I—C bond angle is 90.8 (3)°. Short I⋯O contacts of 2.555 (6) and 2.823 (7) Å are observed due to the strong electrostatic inter­action between two iodo­nium cations and two tri­fluoro­acetate counter-ions (Fig. 2 ▸). There are also C—H⋯F and C—H⋯O inter­actions present (Table 1 ▸). The C—H⋯O inter­actions give rise to two-dimensional sheets in the (001) plane, with the C—H⋯F inter­actions holding the tri­fluoro­acetate anion in place within the sheets (Fig. 3 ▸). The combination of the weak hydrogen bonds with the I⋯O inter­actions gives rise to double-layered sheets, also in the (001) plane. These inter­actions are comparable to those observed in phen­yl(phenyl­ethyn­yl)iodo­nium tosyl­ate and phen­yl(phenyl­ethyn­yl)iodo­nium tri­fluoro­acetate salts (Dixon *et al.*, 2013 ▸).

## Synthesis and crystallization

*m-*CPBA (70% active oxidant, 791 mg, 3.21 mmol, 1.3 eq.) was added to a solution of methyl 4-iodo-3-meth­oxy­isoxazole-5-carboxyl­ate (700 mg, 2.47 mmol, 1.0 eq.) in AcOH (20 ml). After stirring at 55°C for 96 h, water (30 ml) was added to the reaction mixture followed by extraction into DCM (3 × 20 ml). The combined organic layers were washed with a saturated aqueous solution of Na_2_CO_3_ (60 ml), dried over Na_2_SO_4_, filtered and concentrated under reduced pressure to afford a colourless solid, which was used without further purification.

Methyl (4-di­acet­oxy­iodo)-3-meth­oxy­isoxazole-5-carboxyl­ate (281 mg, 0.7 mmol, 1.0 eq.), as a 40% mixture determined by ^1^H-NMR, with methyl 4-iodo-3-meth­oxy­isoxazole-5-carboxyl­ate, was dissolved in DCM (10 ml) and cooled to −30°C, followed by dropwise addition of TFA (110 ml, 1.40 mmol, 2.0 eq.). The solution was stirred with the exclusion of light for 30 min, followed by 1 h at rt. The reaction mixture was re-cooled to −30°C and tribut­yl(4-meth­oxy­phen­yl)stannane (278 mg, 0.70 mmol, 1.0 eq.) added. The reaction was warmed to rt for the second time and left to stir overnight. The solvent was removed *in vacuo*. Upon the addition of Et_2_O, the (3-meth­oxy-5-(meth­oxy­carbon­yl)isoxazol-4-yl)(4-meth­oxy­phen­yl)iodo­nium TFA salt (35 mg, 10%) crystallized. Crystals suitable for X-ray structure determination were obtained from the diffusion of diethyl ether into a dichloromethane solution of the title compound.

^1^H (500 MHz, *d*_6_-DMSO), *δ*: (p.p.m): 8.00 (2*H*, *d*, ^3^ *J*_HH_ 7.2), 7.06 (2*H*, *d*, ^3^ *J*_HH_ 7.2), 4.07 (3*H*, *s*), 4.01 (3*H*, *s*), 3.80 (3*H*, *s*); ^13^C (125 MHz, *d*_6_-DMSO), *δ*: (p.p.m): 170.1, 162.0, 161.0, 155.3, 137.0, 117.5, 107.0, 83.9, 59.0, 55.7, 54.0; ^19^F (470 MHz, *d*_6_-DMSO), *δ*: (p.p.m): −73.6 (3 F, s); HRMS *m*/*z* (ESI^+^), [*M* − TFA]^+^ calculated (C_13_H_13_NO_5_^127^I) 389.9833, found 389.9819.

## Refinement

Crystal data, data collection and structure refinement details are summarized in Table 2 ▸. The maximum residual electron density peak of 1.48 e Å^−3^ was located 1.01 Å from the I4 atom.

## Supplementary Material

Crystal structure: contains datablock(s) global, I. DOI: 10.1107/S2414314623003000/tk4090sup1.cif

Structure factors: contains datablock(s) I. DOI: 10.1107/S2414314623003000/tk4090Isup2.hkl

Supporting information file. DOI: 10.1107/S2414314623003000/tk4090Isup3.mol

Supporting information file. DOI: 10.1107/S2414314623003000/tk4090Isup4.cml

CCDC reference: 2253285

Additional supporting information:  crystallographic information; 3D view; checkCIF report

## Figures and Tables

**Table 1:** Hydrogen-bond geometry (Å, °)

*D*—H⋯*A*	*D*—H	H⋯*A*	*D*⋯*A*	*D*—H⋯*A*
C8—H8*C*⋯O12^i^	0.98	2.58	3.475 (14)	152
C11—H11⋯F16^ii^	0.95	2.56	3.405 (17)	148
C15—H15*C*⋯O6^iii^	0.98	2.62	3.503 (14)	151

Symmetry codes: (i) 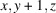 ; (ii) 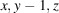 ; (iii) 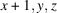 .

**Table 2:** Experimental details

Crystal data	
Chemical formula	C_13_H_13_INO_5_^+^·C_2_F_3_O_2_^−^
*M*_r_	503.17
Crystal system, space group	Triclinic, *P* 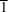
Temperature (K)	173
*a*, *b*, *c* (Å)	8.436 (2), 10.750 (3), 11.338 (3)
α, β, γ (°)	113.913 (5), 97.392 (4), 98.975 (5)
*V* (Å^3^)	907.2 (4)
*Z*	2
Radiation type	Mo *K*α
μ (mm^−1^)	1.83
Crystal size (mm)	0.16 × 0.03 × 0.01

Data collection	
Diffractometer	Rigaku XtaLAB P200
Absorption correction	Multi-scan (*CrystalClear*; Rigaku, 2014 ▸)
*T*_min_, *T*_max_	0.862, 0.982
No. of measured, independent and observed [*F* ^2^ > 2.0σ(*F* ^2^)] reflections	11079, 3270, 2423
*R*_int_	0.053
(sin θ/λ)_max_ (Å^−1^)	0.603

Refinement	
*R*[*F* ^2^ > 2σ(*F* ^2^)], *wR*(*F* ^2^), *S*	0.060, 0.145, 1.04
No. of reflections	3270
No. of parameters	246
H-atom treatment	H-atom parameters constrained
Δρ_max_, Δρ_min_ (e Å^−3^)	1.49, −0.65

Computer programs: *CrystalClear* (Rigaku, 2014 ▸), *DIRDIF99* (Beurskens *et al.*, 1999 ▸), *SHELXL2018/3* (Sheldrick, 2015 ▸), *OLEX2* (Dolomanov *et al.*, 2009 ▸), *Mercury* (Macrae *et al.*, 2020 ▸), *CrystalStructure* (Rigaku, 2018 ▸), *enCIFer* (Allen *et al.*, 2004 ▸) and *publCIF* (Westrip, 2010 ▸).

## full crystallographic data

### Crystal data

**Table:**

C_13_H_13_INO_5_^+^·C_2_F_3_O_2_^−^	*Z* = 2
*M**_r_* = 503.17	*F*(000) = 492.00
Triclinic, *P*1	*D*_x_ = 1.842 Mg m^−^^3^
*a* = 8.436 (2) Å	Mo *K*α radiation, λ = 0.71073 Å
*b* = 10.750 (3) Å	Cell parameters from 1722 reflections
*c* = 11.338 (3) Å	θ = 2.0–25.3°
α = 113.913 (5)°	µ = 1.83 mm^−^^1^
β = 97.392 (4)°	*T* = 173 K
γ = 98.975 (5)°	Plate, colourless
*V* = 907.2 (4) Å^3^	0.16 × 0.03 × 0.01 mm

### Data collection

**Table:**

Rigaku XtaLAB P200 diffractometer	3270 independent reflections
Radiation source: Rotating Anode, Rigaku FR-X	2423 reflections with *F*^2^ > 2.0σ(*F*^2^)
Rigaku Osmic Confocal Optical System monochromator	*R*_int_ = 0.053
Detector resolution: 5.814 pixels mm^-1^	θ_max_ = 25.4°, θ_min_ = 2.0°
ω scans	*h* = −10→10
Absorption correction: multi-scan (CrystalClear; Rigaku, 2014)	*k* = −12→12
*T*_min_ = 0.862, *T*_max_ = 0.982	*l* = −13→13
11079 measured reflections	

### Refinement

**Table:**

Refinement on *F*^2^	Secondary atom site location: difference Fourier map
*R*[*F*^2^ > 2σ(*F*^2^)] = 0.060	Hydrogen site location: inferred from neighbouring sites
*wR*(*F*^2^) = 0.145	H-atom parameters constrained
*S* = 1.04	*w* = 1/[σ^2^(*F*_o_^2^) + (0.0635*P*)^2^ + 4.1326*P*] where *P* = (*F*_o_^2^ + 2*F*_c_^2^)/3
3270 reflections	(Δ/σ)_max_ < 0.001
246 parameters	Δρ_max_ = 1.49 e Å^−^^3^
0 restraints	Δρ_min_ = −0.65 e Å^−^^3^
Primary atom site location: structure-invariant direct methods	

### Special details

**Table:**

Geometry. All esds (except the esd in the dihedral angle between two l.s. planes) are estimated using the full covariance matrix. The cell esds are taken into account individually in the estimation of esds in distances, angles and torsion angles; correlations between esds in cell parameters are only used when they are defined by crystal symmetry. An approximate (isotropic) treatment of cell esds is used for estimating esds involving l.s. planes.
Refinement. Carbon-bound H atoms were included in calculated positions (C—H = 0.95–0.98 Å) and refined as riding atoms with *U*_iso_(H) = 1.2*U*_eq_ (*sp*^2^) or *U*_iso_(H) = 1.5*U*_eq_ (*sp*^3^).

### Fractional atomic coordinates and isotropic or equivalent isotropic displacement parameters (Å^2^)

**Table:**

	*x*	*y*	*z*	*U*_iso_*/*U*_eq_	
I4	0.20970 (6)	0.42981 (5)	0.12013 (5)	0.0509 (2)	
F16	0.2459 (9)	0.9270 (9)	0.2807 (8)	0.152 (4)	
F17	0.0378 (8)	0.9854 (6)	0.3427 (6)	0.111 (3)	
F18	0.1490 (12)	0.8548 (8)	0.4008 (6)	0.136 (3)	
O1	0.4332 (8)	0.6834 (6)	0.5238 (5)	0.0632 (16)	
O3	0.5054 (8)	0.7371 (7)	0.2570 (7)	0.0717 (18)	
O6	0.1162 (9)	0.3812 (7)	0.3838 (7)	0.0777 (19)	
O7	0.2682 (9)	0.4996 (8)	0.5853 (7)	0.081 (2)	
O12	0.7116 (10)	0.0695 (8)	0.1381 (8)	0.094 (2)	
O17	−0.0913 (8)	0.7775 (6)	0.1053 (6)	0.076 (2)	
O18	0.0342 (9)	0.6423 (7)	0.1656 (7)	0.086 (2)	
N2	0.5171 (10)	0.7647 (9)	0.4715 (8)	0.074 (2)	
C3	0.4577 (11)	0.6993 (9)	0.3442 (9)	0.061 (3)	
C4	0.3342 (10)	0.5742 (9)	0.3109 (8)	0.057 (2)	
C5	0.3256 (11)	0.5714 (10)	0.4265 (9)	0.064 (2)	
C6	0.2232 (12)	0.4716 (11)	0.4574 (9)	0.064 (3)	
C7	0.1665 (14)	0.4074 (13)	0.6255 (13)	0.095 (4)	
H7A	0.165550	0.309983	0.568918	0.143*	
H7B	0.054106	0.421058	0.616962	0.143*	
H7C	0.211381	0.429339	0.717581	0.143*	
C8	0.6252 (12)	0.8788 (11)	0.3162 (11)	0.084 (3)	
H8A	0.723922	0.876368	0.370487	0.126*	
H8B	0.573436	0.950570	0.371279	0.126*	
H8C	0.655593	0.900916	0.245180	0.126*	
C9	0.3799 (11)	0.3064 (9)	0.1113 (8)	0.055 (2)	
C10	0.3599 (15)	0.2162 (11)	0.1705 (9)	0.080 (3)	
H10	0.267985	0.210169	0.209962	0.096*	
C11	0.4686 (15)	0.1368 (12)	0.1732 (10)	0.083 (3)	
H11	0.447999	0.068052	0.205282	0.099*	
C12	0.5986 (13)	0.1563 (11)	0.1318 (11)	0.070 (3)	
C13	0.6360 (12)	0.2401 (12)	0.0699 (11)	0.090 (4)	
H13	0.733464	0.245200	0.036741	0.108*	
C14	0.5113 (13)	0.3240 (11)	0.0579 (10)	0.077 (3)	
H14	0.524458	0.384409	0.015910	0.093*	
C15	0.8561 (13)	0.1021 (16)	0.1053 (14)	0.137 (7)	
H15A	0.925082	0.038937	0.112025	0.206*	
H15B	0.835017	0.092238	0.014517	0.206*	
H15C	0.912545	0.198818	0.165580	0.206*	
C16	0.1083 (10)	0.8821 (9)	0.3002 (8)	0.056 (2)	
C17	0.0083 (9)	0.7542 (9)	0.1774 (8)	0.049 (2)	

### Atomic displacement parameters (Å^2^)

**Table:**

	*U*^11^	*U*^22^	*U*^33^	*U*^12^	*U*^13^	*U*^23^
I4	0.0529 (4)	0.0449 (3)	0.0430 (3)	0.0166 (2)	−0.0062 (2)	0.0102 (2)
F16	0.085 (5)	0.135 (7)	0.130 (7)	−0.041 (5)	0.028 (5)	−0.023 (5)
F17	0.099 (5)	0.068 (4)	0.098 (5)	0.038 (4)	−0.031 (4)	−0.021 (3)
F18	0.212 (9)	0.098 (5)	0.057 (4)	0.025 (5)	−0.042 (5)	0.017 (4)
O1	0.067 (4)	0.062 (4)	0.040 (3)	0.014 (3)	−0.004 (3)	0.007 (3)
O3	0.069 (4)	0.068 (4)	0.077 (5)	0.009 (3)	0.009 (4)	0.035 (4)
O6	0.075 (5)	0.071 (5)	0.073 (4)	−0.005 (4)	0.003 (4)	0.029 (4)
O7	0.080 (5)	0.101 (5)	0.063 (4)	0.023 (4)	0.009 (4)	0.037 (4)
O12	0.105 (6)	0.095 (6)	0.093 (5)	0.038 (5)	0.019 (5)	0.047 (5)
O17	0.089 (5)	0.054 (4)	0.060 (4)	0.020 (3)	−0.024 (3)	0.011 (3)
O18	0.096 (5)	0.057 (4)	0.083 (5)	0.032 (4)	−0.019 (4)	0.013 (4)
N2	0.070 (5)	0.067 (5)	0.068 (5)	0.006 (4)	−0.002 (4)	0.019 (5)
C3	0.052 (5)	0.046 (5)	0.054 (5)	0.012 (4)	−0.015 (4)	−0.002 (4)
C4	0.057 (5)	0.060 (6)	0.045 (5)	0.027 (5)	0.000 (4)	0.011 (4)
C5	0.056 (5)	0.065 (6)	0.055 (5)	0.019 (5)	0.002 (4)	0.013 (5)
C6	0.058 (6)	0.069 (7)	0.057 (6)	0.027 (5)	−0.002 (5)	0.021 (5)
C7	0.078 (8)	0.118 (10)	0.118 (10)	0.029 (7)	0.036 (7)	0.073 (9)
C8	0.065 (6)	0.067 (7)	0.091 (8)	−0.002 (5)	−0.010 (5)	0.020 (6)
C9	0.059 (5)	0.051 (5)	0.043 (4)	0.023 (4)	−0.006 (4)	0.010 (4)
C10	0.115 (9)	0.073 (7)	0.051 (5)	0.054 (6)	0.009 (5)	0.016 (5)
C11	0.099 (9)	0.090 (8)	0.062 (6)	0.044 (7)	0.016 (6)	0.028 (6)
C12	0.057 (6)	0.083 (7)	0.079 (7)	0.012 (5)	0.002 (5)	0.051 (6)
C13	0.055 (6)	0.083 (8)	0.078 (7)	0.000 (6)	0.025 (5)	−0.015 (6)
C14	0.074 (7)	0.060 (6)	0.079 (7)	0.014 (5)	0.025 (6)	0.010 (5)
C15	0.042 (6)	0.154 (13)	0.122 (11)	−0.036 (7)	0.039 (7)	−0.018 (9)
C16	0.043 (5)	0.057 (6)	0.051 (5)	0.008 (4)	−0.003 (4)	0.013 (4)
C17	0.043 (4)	0.048 (5)	0.047 (5)	0.018 (4)	0.005 (4)	0.012 (4)

### Geometric parameters (Å, º)

**Table:**

I4—C9	2.087 (8)	C7—H7A	0.9800
I4—C4	2.092 (8)	C7—H7B	0.9800
F16—C16	1.268 (11)	C7—H7C	0.9800
F17—C16	1.290 (10)	C8—H8A	0.9800
F18—C16	1.307 (11)	C8—H8B	0.9800
O1—C5	1.347 (10)	C8—H8C	0.9800
O1—N2	1.397 (10)	C9—C14	1.353 (13)
O3—C3	1.297 (12)	C9—C10	1.388 (14)
O3—C8	1.520 (11)	C10—C11	1.353 (14)
O6—C6	1.154 (11)	C10—H10	0.9500
O7—C6	1.344 (11)	C11—C12	1.268 (15)
O7—C7	1.459 (13)	C11—H11	0.9500
O12—C15	1.355 (12)	C12—C13	1.374 (15)
O12—C12	1.449 (12)	C13—C14	1.515 (16)
O17—C17	1.221 (9)	C13—H13	0.9500
O18—C17	1.214 (10)	C14—H14	0.9500
N2—C3	1.308 (12)	C15—H15A	0.9800
C3—C4	1.445 (13)	C15—H15B	0.9800
C4—C5	1.334 (13)	C15—H15C	0.9800
C5—C6	1.453 (14)	C16—C17	1.524 (11)
			
C9—I4—C4	90.8 (3)	C10—C9—I4	117.9 (7)
C5—O1—N2	110.3 (7)	C11—C10—C9	121.3 (11)
C3—O3—C8	113.8 (8)	C11—C10—H10	119.3
C6—O7—C7	114.1 (9)	C9—C10—H10	119.3
C15—O12—C12	114.6 (11)	C12—C11—C10	118.4 (12)
C3—N2—O1	104.8 (8)	C12—C11—H11	120.8
O3—C3—N2	125.4 (9)	C10—C11—H11	120.8
O3—C3—C4	123.4 (8)	C11—C12—C13	127.0 (11)
N2—C3—C4	111.1 (10)	C11—C12—O12	116.1 (10)
C5—C4—C3	104.5 (8)	C13—C12—O12	116.5 (10)
C5—C4—I4	129.6 (8)	C12—C13—C14	115.5 (9)
C3—C4—I4	125.8 (7)	C12—C13—H13	122.2
C4—C5—O1	109.2 (9)	C14—C13—H13	122.2
C4—C5—C6	130.6 (9)	C9—C14—C13	115.1 (10)
O1—C5—C6	120.2 (9)	C9—C14—H14	122.5
O6—C6—O7	123.9 (10)	C13—C14—H14	122.5
O6—C6—C5	125.4 (9)	O12—C15—H15A	109.5
O7—C6—C5	110.8 (9)	O12—C15—H15B	109.5
O7—C7—H7A	109.5	H15A—C15—H15B	109.5
O7—C7—H7B	109.5	O12—C15—H15C	109.5
H7A—C7—H7B	109.5	H15A—C15—H15C	109.5
O7—C7—H7C	109.5	H15B—C15—H15C	109.5
H7A—C7—H7C	109.5	F16—C16—F17	107.8 (9)
H7B—C7—H7C	109.5	F16—C16—F18	103.2 (9)
O3—C8—H8A	109.5	F17—C16—F18	105.7 (8)
O3—C8—H8B	109.5	F16—C16—C17	110.8 (8)
H8A—C8—H8B	109.5	F17—C16—C17	115.2 (7)
O3—C8—H8C	109.5	F18—C16—C17	113.2 (8)
H8A—C8—H8C	109.5	O18—C17—O17	128.3 (8)
H8B—C8—H8C	109.5	O18—C17—C16	116.1 (7)
C14—C9—C10	122.3 (9)	O17—C17—C16	115.6 (7)
C14—C9—I4	119.7 (7)		
			
C5—O1—N2—C3	0.0 (9)	O1—C5—C6—O7	−7.7 (11)
C8—O3—C3—N2	−7.7 (13)	C14—C9—C10—C11	−2.6 (15)
C8—O3—C3—C4	174.8 (8)	I4—C9—C10—C11	−177.5 (8)
O1—N2—C3—O3	−177.8 (8)	C9—C10—C11—C12	6.9 (16)
O1—N2—C3—C4	−0.1 (10)	C10—C11—C12—C13	−7.7 (18)
O3—C3—C4—C5	177.9 (8)	C10—C11—C12—O12	179.4 (9)
N2—C3—C4—C5	0.1 (10)	C15—O12—C12—C11	−173.9 (10)
O3—C3—C4—I4	0.4 (12)	C15—O12—C12—C13	12.4 (14)
N2—C3—C4—I4	−177.4 (6)	C11—C12—C13—C14	3.9 (17)
C3—C4—C5—O1	−0.1 (10)	O12—C12—C13—C14	176.8 (8)
I4—C4—C5—O1	177.3 (5)	C10—C9—C14—C13	−1.1 (13)
C3—C4—C5—C6	179.6 (9)	I4—C9—C14—C13	173.7 (6)
I4—C4—C5—C6	−3.0 (15)	C12—C13—C14—C9	0.7 (13)
N2—O1—C5—C4	0.1 (10)	F16—C16—C17—O18	86.1 (11)
N2—O1—C5—C6	−179.7 (8)	F17—C16—C17—O18	−151.2 (9)
C7—O7—C6—O6	−1.6 (14)	F18—C16—C17—O18	−29.4 (12)
C7—O7—C6—C5	177.5 (8)	F16—C16—C17—O17	−96.1 (11)
C4—C5—C6—O6	−8.3 (16)	F17—C16—C17—O17	26.7 (12)
O1—C5—C6—O6	171.4 (9)	F18—C16—C17—O17	148.5 (9)
C4—C5—C6—O7	172.5 (9)		

### Hydrogen-bond geometry (Å, º)

**Table:**

*D*—H···*A*	*D*—H	H···*A*	*D*···*A*	*D*—H···*A*
C8—H8*C*···O12^i^	0.98	2.58	3.475 (14)	152
C11—H11···F16^ii^	0.95	2.56	3.405 (17)	148
C15—H15*C*···O6^iii^	0.98	2.62	3.503 (14)	151

Symmetry codes: (i) *x*, *y*+1, *z*; (ii) *x*, *y*−1, *z*; (iii) *x*+1, *y*, *z*.
